# A Novel Rain Removal Approach for Outdoor Dynamic Vision Sensor Event Videos

**DOI:** 10.3389/fnbot.2022.928707

**Published:** 2022-08-04

**Authors:** Long Cheng, Ni Liu, Xusen Guo, Yuhao Shen, Zijun Meng, Kai Huang, Xiaoqin Zhang

**Affiliations:** ^1^College of Computer Science and Artificial Intelligence, Wenzhou University, Wenzhou, China; ^2^School of Computer Science and Engineering, Sun Yat-sen University, Guangzhou, China; ^3^Key Laboratory of Machine Intelligence and Advanced Computing, Guangzhou, China

**Keywords:** dynamic vision sensors, rain removal, intelligent driving, outdoor vision systems, deraining

## Abstract

As bio-inspired vision devices, dynamic vision sensors (DVS) are being applied in more and more applications. Unlike normal cameras, pixels in DVS independently respond to the luminance change with asynchronous output spikes. Therefore, removing raindrops and streaks from DVS event videos is a new but challenging task as the conventional deraining methods are no longer applicable. In this article, we propose to perform the deraining process in the width and time (W-T) space. This is motivated by the observation that rain steaks exhibits discontinuity in the width and time directions while background moving objects are usually piecewise smooth along with both directions. The W-T space can fuse the discontinuity in both directions and thus transforms raindrops and streaks to approximately uniform noise that are easy to remove. The non-local means filter is adopted as background object motion has periodic patterns in the W-T space. A repairing method is also designed to restore edge details erased during the deraining process. Experimental results demonstrate that our approach can better remove rain noise than the four existing methods for traditional camera videos. We also study how the event buffer depth and event frame time affect the performance investigate the potential implementation of our approach to classic RGB images. A new real-world database for DVS deraining is also created and shared for public use.

## 1. Introduction

Recently, the bio-inspired Dynamic Vision Sensors (DVS) are becoming more and more popular in vision applications due to the fast response time, ability of adaptive sensing in rapidly changing light conditions, and low power consumption (Soman et al., 2016). Pixels in DVS independently respond to the luminance change with asynchronous output spikes by mimicking the neural architectures present in biologic nervous systems. The speed of a DVS is not limited by traditional concepts such as exposure time. Hence, it can capture fast motion which can only be detected by expensive high-speed cameras. Moreover, a DVS also has attractive features such as high dynamic range, high temporal resolution, and little redundant information. All of these make DVS a promising vision sensor for the applications in wearable devices, intelligent driving, and outdoor surveillance (Bi et al., 2018; Chen et al., 2020). Particularly, since DVS has a strong resistance to artifacts from flickering light sources such as the LED traffic signs and car taillights, it is expected that DVS has a significant impact on the rapid analysis or real-time monitoring of intelligent driving applications (Dong et al., 2019).

Designers are facing a serious problem during implementing the DVS for outdoor vision applications. The sensors are frequently affected by various bad weather conditions (Li et al., 2016), especially by rain. Raindrops inevitably cause dynamic streaks at high velocities on the acquired images. These rain streaks may deform and interfere with nearby regions and, hence, degrade the performance of many algorithms of the DVS vision systems such as tracking, recognition, and object detection. Therefore, to improve the performance of such applications in rainy conditions, the rain streaks outputted from a DVS should be eliminated.

For traditional cameras, there have been plenty of studies on this topic. Garg and Nayar (2004) first exploited the dynamic motion of raindrops with irradiance constraints to remove rain streaks from videos. Since then, researchers have proposed many methods that are based on the priors of rain streaks on photometric appearance (Shen and Xue, 2011; Tripathi and Mukhopadhyay, 2011), frequency domain (Barnum et al., 2007, 2010), repetitive and discontinuous local patterns (Li M. et al., 2018), temporal correlations (Kim J.-H. et al., 2015), joint spatial and wavelet domain features (Xue et al., 2012; Zhang et al., 2019), and spatial discriminatively (Jiang et al., 2017). Moreover, methods depending on the matrix decomposition (Ren et al., 2017), Gaussian Mixture Model (GMM) distribution (Chen and Chau, 2013), and the low-rank property of rain free scenes (Abdel-Hakim, 2014; Kim J.-H. et al., 2015) have also been presented.

While having made great improvements to the field, current rain removal approaches are no longer suitable for DVS. Directly applying them may cause low efficiency, serious blurs, or even fails in detecting and removing rain streaks. It is attributed to the factors in two aspects. First, the RGB and grayscale information, which are common in traditional cameras, are not presented in DVS data since a DVS only produces streams of events. Second, due to the strong response to light intensity change, the visual appearance of rain streaks becomes much more evident in DVS, which is a scene different from traditional rainy images. Removing rain streaks from data outputted from DVS cameras is a new and important problem that deserves more researchers' attention.

In this article, we focus on addressing a such problem and present a novel, simple but surprisingly effective approach to removing rain streaks from videos acquired from a stationary DVS camera. The idea of our approach comes from the observation that raindrops are usually small-sized water droplets having higher velocities along the vertical direction. This indicates two intrinsic properties of raindrops and streaks on images: the discontinuity in the width direction due to their small sizes and motion directions closer to the vertical direction, and the discontinuity in the temporal direction. In DVS cameras, such properties are more distinctive since the visual appearance of raindrops is more evident. Therefore, we propose to view and process the DVS images from the *width and time (W-T) perspective* instead of the normal height and width perspective. In the W-T perspective, we can fuse the discontinuity in the width and temporal directions and thus transform the rain into approximately uniform noise which can be easily removed. The contributions of this article can be summarized as follows.

- Width and Time Space Deraining (WTSD) model. We present the first DVS image deraining model where the rain steaks are detected and removed in the W-T space, which significantly reinforces the discontinuity of rain streaks. In the W-T space, rain streaks usually have different geometric and temporal properties, compared with the background scene, thus can be removed by adopting de-noising algorithms. To restore the useful details erased during the denoising process, we combine the images before and after the deraining process and also present a method to retrieve the source information for the erased parts.
- DVS-Deraining (DVS-D) database. We create a DVS-D database which collects a variety of dynamic vision sensor rain images and their derained ground truth. To the best of our knowledge, this is the first real-world deraining database for Dynamic Vision Sensor outputs.
- We conduct a set of systematic experiments to investigate the effectiveness of our approach. The relationship between approach parameters and performance is studied comprehensively. Moreover, three traditional approaches are also implemented for comparison. The results demonstrate that our approach can not only remove most rain streaks for DVS videos in an online manner but also has the potential to handle traditional camera cases.

The rest of this article is organized as follows. Section 2 discusses the related studies. We discuss the working principles of DVS cameras and analyze the differences between images produced by a DVS and a traditional camera in Section 3. Then, we introduce the discontinuity of rain streaks in Section 4. Section 5 presents our proposed approach. Experiments results are shown in Section 6. Section 7 concludes this article.

## 2. Related Study

We briefly introduce existing rain removal studies on the traditional camera platforms.

To quantitatively measure the quality of the derained image, Wu et al. (2020) propose a bi-directional feature embedding network (B-FEN) and also create a deraining quality assessment (DQA) database. For the problem of single image deraining, Zhang and Patel (2017) utilize a set of convolutional filters to represent background clear images and rain streaks, respectively. Once these filters have been learned, the rainy image can be decomposed by solving an optimization problem. In Kang et al. (2011), a rainy image is decomposed into low frequency and high frequency parts by a bilateral filter. Rain streaks in the high frequency part are separated by morphological component analysis (MCA)-based dictionary learning and sparse coding. Noted the limitations of standard batch-mode learning-based approaches, Sun et al. (2014) present a novel rain removal method that utilizes the structural similarity of the image bases. The Gaussian mixture model (GMM) is exploited to patch priors of rain streaks with various scales in Li et al. (2016, 2017). Moreover, promising deep learning and artificial neural networks have also been studied for this problem (Li X. et al., 2018; Liu et al., 2018; Qian et al., 2018; Zhang and Patel, 2018; Jiang et al., 2020). Zhu et al. (2020) propose a gated non-local deep residual learning framework that can avoid over-deraining or under-deraining. In Ding et al. (2021), studied to remove rain streaks in light field images according to the observation that they usually have different slopes and/or chromatic values, compared with the background scene, along with the epipolar plane images. Since a single image provides less information than a video, these approaches usually suffer from high computation requirements or limited performance and thus may not be suitable for the online implementation of DVS deraining.

For the video rain removal task, pioneering research is presented in Garg and Nayar (2004). The visual appearance of raindrops on vision systems and the photometric model are studied. Then, rain streaks are detected by combining the linear photometric constraint and their dynamic motions. This approach may generate false detections between rain streaks and moving objects (Kim H. G. et al., 2015) and the linear photometric constraint is also not valid for DVS platforms. Zhang et al. propose to exploit both temporal and chromatic properties of rain streaks to detect them in the video. Images often get blurred due to a temporal average of the background. In addition, the chromatic property no longer exists for rain streaks in DVS. Barnum et al. analyze the overall effect of rain and snow in the frequency domain by using a physical and statistical model (Barnum et al., 2007, 2010). The amount of rain or snow can be reduced by suppressing the detected rain or snow parts in the frequency domain. This approach gives false detection results when frequencies corresponding to rain are too cluttered (Tripathi and Mukhopadhyay, 2014). Hiroyuki et al. propose to remove snowfall-liked noise in an image sequence by applying the temporal median filter to each pixel of the successive images. While this method is extremely fast for online implementation, any movement will cause blurring artifacts (Barnum et al., 2007). Besides, filtering the noise only along the temporal direction limits its performance. Additional early methods on this topic have been summarized in Tripathi and Mukhopadhyay (2014).

An important aspect of DVS rain removal is object detection and tracking. Zhang et al. (2015) proposed a robust and general tracking system by using dominant color-spatial based object representation and bin-ratio based similarity measure. In Lee and Wittenburg (2019), proposed to perform object detection by video slicing. Although the Width and Time space are similar to video slicing, our study differs from Lee and Wittenburg (2019) in two aspects: (1) deraining vs. object detection; (2) DVS vs. RGB camera. In terms of video slicing, the main contributions are: (1) we fully and systematically investigate how the shape of a rain streak changes after slicing and present two observations. (2) In the experiment, we study how the event buffer depth, a parameter of video slicing, affects the performance of deraining. We found that event buffer depth has little influence on the deraining results.

Removing rain from DVS event videos is a newly emerged issue that is essential to the applications designed for DVS. People have paid little attention to this topic and we can hardly find any related research for DVS. To the best of our knowledge, this article is the first one focusing on the problem of deraining event videos from a stationary DVS camera.

## 3. Dynamic Vision Sensors

A CeleX^TM^ DVS is adopted as an example platform. We first introduce its basic working principles and output data. Then, we summarize the unique features of DVS images.

### 3.1. The Basic Working Principle

**Figure 1:**
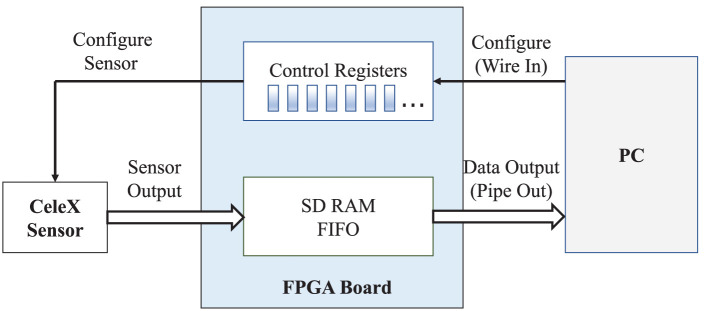
The working principle of the CeleX^TM^ chipset.

A CeleX DVS is highly efficient with a customized FPGA board as the interface for configuring and reading output data. Applications could configure the sensor by setting the control registers in the FPGA. Sensor output data is also handled by the FPGA and eventually output to the application according to the configuration, as demonstrated in Figure 1.

The essential difference between a DVS and a traditional camera is that each pixel in a DVS can individually monitor the relative change in light intensity and autonomously generate an output event. A CeleX DVS has asynchronous row and column arbitration circuits to process these output requests and ensure only one is granted at a time when they receive multiple simultaneous requests (CeleX4, 2019). Therefore, the output of a DVS is not a classical frame, but a stream of asynchronous digital events.

A CeleX sensor provided with the customized FPGA board and corresponding Software Development Kit (SDK) has three working modes: *Full-Picture data, Event Data*, and *Optical-Flow data*. The *Full-Picture data* mode outputs the information of all pixels sequentially, which is similar to conventional cameras. When the *Event Data* mode is enabled, the sensor only outputs the values of active pixels the intensity change at which exceeds the predefined threshold. In the *Optical-Flow data* mode, the SDK outputs optical flow data, i.e., the speed and direction of each pixel.

In this article, we focus on rain removal for DVS working in the *Event Data* mode. One can also base the optical flow data on the detection and removing rain. However, since raindrops distribute randomly in 3D space, their projection of them onto the sensor has different velocities and intensities. The sensor in *Optical-Flow data* mode can hardly capture the accurate motion of all rain streaks, particularly those very thin or short. On the contrary, these rain streaks are well-sampled in the *Event Data*. An event from a CeleX DVS can be specified by a tuple (*X*, *Y*, *A*, *T*), where *X* and *Y* are the address (row and column) of the pixel triggering an event, and *A* and *T* are the absolute brightness and activation time when the pixel event is triggered, respectively. In the *Event Data* mode, the events of object motion are grouped as event frames and outputted periodically, the Event Frame Time can be adjusted from 1 to 1,000 ms.

### 3.2. Unique Features

According to the working principle of DVS cameras, the data produced has the following unique features compared to that from traditional cameras.

(A) No information about the stationary background is preserved and only object motions are captured.
(B) The output event images are approximately binary ones, i.e., no grayscale OR RGB information.

The first feature benefits rain removal due to two reasons. First, the rain streaks are evident since only object motions are captured. Second, we only need to separate rain streaks from moving objects, as the stationary background is not presented. However, the second feature also poses challenges to the rain detection process because of the loss of grayscale and RGB information, which deliver several important constraints that have been widely exploited to detect rain streaks.

**Figure 2:**
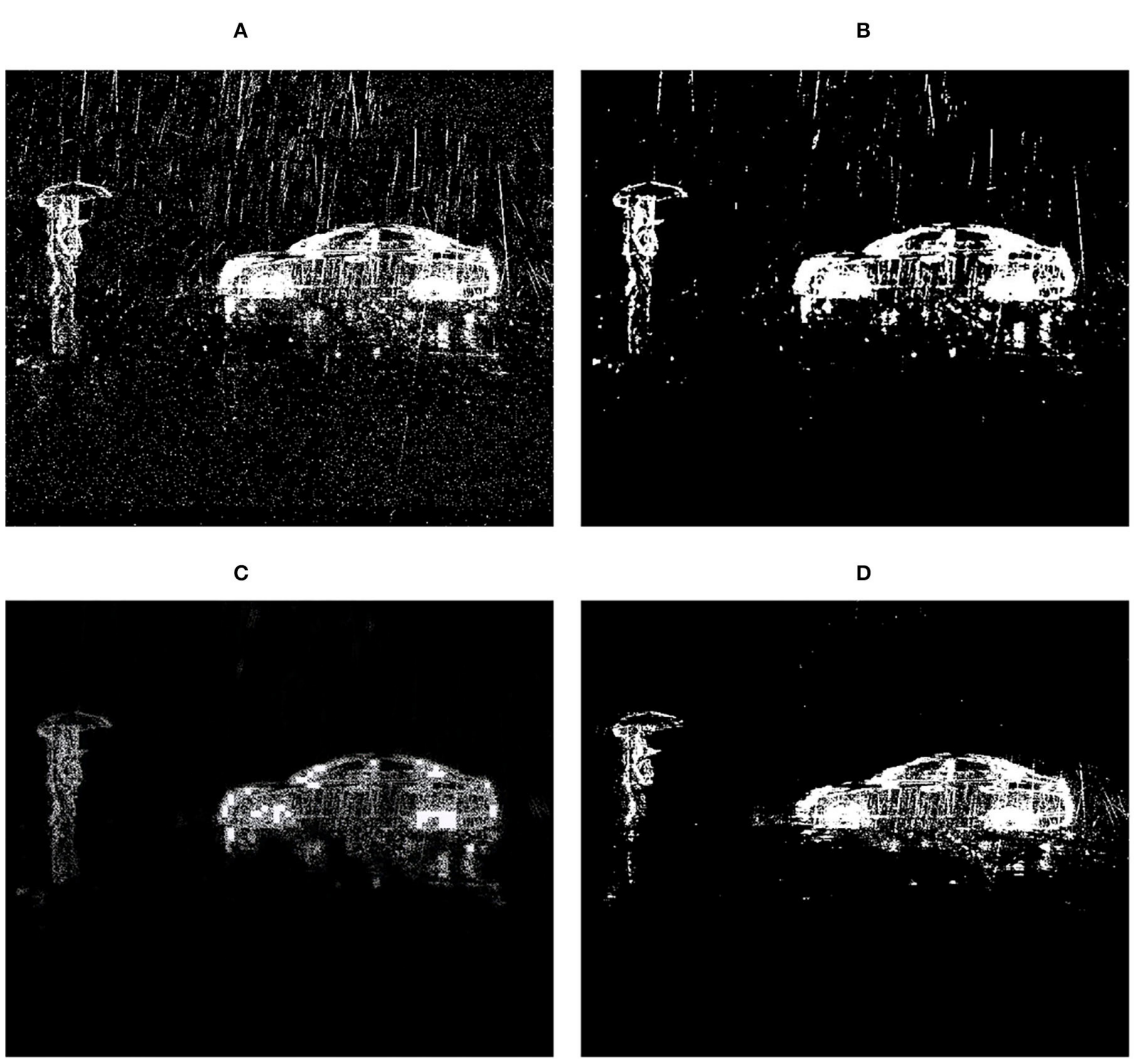
A rainy DVS frame and the deraining results of two existing algorithms and our approach. Observe MCSC (Li M. et al., 2018) only removes part of the rain streaks and ReMAEN (Yang and Lu, 2019) failed to preserve most texture details of background objects. **(A)** Input. **(B)** MCSC. **(C)** ReMAEN. **(D)** Our.

Because of these features, the traditional camera-oriented deraining approaches may produce pessimistic results when handling DVS data. Examples are shown in Figure 2. On the one hand, new mechanisms are demanded to separate rain streaks and background objects without using the grayscale and RGB information. On the other hand, we should also study and utilize the feature (A), which has not been considered during the deraining process in existing methods. Therefore, removing rain streaks from data outputted from DVS cameras is an important problem. A new DVS-camera oriented deraining approach is demanded.

## 4. Prior Knowledge

In general, the observation model for a rainy video can be termed O, where O is a 3-order tensor representing the input video. Here, *h* × *w* is the event frame size (height × width) and *t* is the time length. The goal of our approach is to distinguish B from O. This is an ill-posed inverse problem (Jiang et al., 2018), which could be handled with prior knowledge.

### 4.1. Discontinuity in Width and Time Directions

Being aware of the loss of RGB and grayscale information from DVS, we should exploit their strong response to motions for deraining. As aforementioned in Section 3.2, the motion of raindrops and streaks are more evident to be recognized. Therefore, we focus on utilizing the dynamic features of rain.

Rain is a collection of randomly distributed water droplets of different shapes and sizes that fall at high velocities (Garg and Nayar, 2004). The size of a raindrop typically varies from 0.1 to 3.0 mm and can be approximately described by the uniform distribution (Barnum et al., 2007). The terminal velocity *v* of a drop is related to its diameter *r*_*d*_ and can be given by $v\left(r_{d}\right)=-0.2+5.0r_{d}-0.9{r_{d}}^{2}+0.1{r_{d}}^{3}$ (Barnum et al., 2007), which could reach about 9.4 m/s for raindrops with a size of 3.0 mm. Compared to normal moving objects, raindrops have the following characteristics: small size, high velocity, and motion directions close to the vertical axis.

Consider a DVS camera observing a volume of rain. A raindrop will be imaged as a straight streak with constant breadth (Barnum et al., 2007). From the above characteristics, one can recognize two intrinsic properties of the rain streaks, i.e., uniform distribution for the breadth *b* and length *l*, and the discontinuity in the width and time directions.

First, *b* and *l* can be predicted according to the diameter *r*_*d*_ of the drop, its distance *z* from the camera, the exposure time *e*, and the focal length *f* (Barnum et al., 2007), i.e., $b\left(r_{d},z\right)=\frac{r_{d}f}{z}$ and $l\left(r_{d},z\right)=\frac{v\left(r_{d}\right)ef}{z}+\frac{r_{d}f}{z}$. It's usually assumed that the distribution of raindrops in the world is uniform and remains constant over time (Garg and Nayar, 2004). Thus, *z* is also uniformly distributed. Then, with fixed focal length and camera speed, the breadth *b* and length *l* have a uniform distribution over space and time. This property is adopted to analyze the transformation of rain streaks in Section 5.1. For the second property, we can consider streaks are discontinuous in the width direction because (1) rain streaks usually occupy only several pixels in the width direction due to their small sizes; (2) their motion directions are generally close to the vertical axis. It is easy to comprehend the discontinuity in time directions because of their small sizes and high velocities. Normally, the probability that a pixel is continuously projected by raindrops for a series of sampling instances is negligible (Garg and Nayar, 2004). One can also figure out such properties from the example shown in **Figure 4**.

Now, we have noticed that rain streaks are discontinuous in the width and time directions, and their breadth and length are also uniformly distributed. For the normal background objects, it has been recognized that they are largely piecewise smooth from the spatial and temporal perspective (Jiang et al., 2018). Therefore, we can utilize such properties to separate background objects from rainy DVS videos, which is discussed in the next section.

## 5. Our Approach

### 5.1. The Width and Time Space

Def. 1 (W-T Space): For a DVS event video specified by a 3-order tensor $O\in {\mathbb{R}}^{h\times w\times t}$, it is viewed as a set of images [*I*_1_, *I*_2_, ⋯, *I*_*t*_] where $I_{i}=O\left(:,:,i\right)$ in the normal space. O(:,:,i) is a two-dimensional section slice of O. In the W-T space, the video is viewed as another set of images $\left[{Î}_{1},{\hat{I}}_{2},\cdots \;,{Î}_{h}\right]$ where ${Î}_{i}=O\left(i,:,:\right)$ and ${Î}_{i}\in {\mathbb{R}}^{w\times t}$. Similarly, O(i,:,:) is the first frontal slice of O.

In the normal space, images are viewed along the time direction while in the W-T space they are viewed along the height direction. An example is displayed in **Figure 4E**. We call the images in the normal and W-T space the *original* and *transformed* images, respectively. When viewed from a different perspective, the appearance of rain motion in the W-T space is no longer streaks.

### 5.2. Rain Streaks in the W-T Space

In this section, we discuss how the projections of rain streaks differ from those in the normal space.

**Figure 3:**
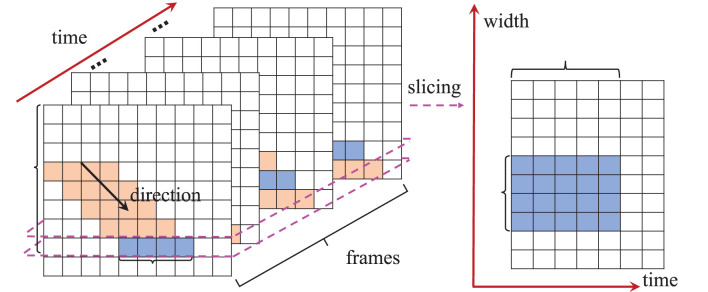
Transformation from normal space to the W-T space.

Consider a DVS camera observing an object moving at a constant velocity and the projection of the object onto the original imaging system is a parallelogram occupying *l* and *b* pixels in the height and width direction, respectively, as shown in Figure 3. We slice the original images at a certain height *H*_*s*_ and obtain the corresponding transformed image $O\left(H_{s},:,:\right)$ in the W-T space. *N*_*p*_ denotes the number of original frames in which the object covers at least one pixel at height *H*_*s*_. Then, the width of the object's projection in the transformed image equals *N*_*p*_. Therefore, the ratio between the object's projection pixel numbers in the W-T space and the normal space is:


(1) $$\begin{array}{r} r_{p}=\frac{b\times N_{p}}{l\times b}=\frac{N_{p}}{l}. \end{array}$$

We approximate the projection of raindrops in the normal space as a parallelogram. When a certain pixel is covered by rain, the probability that its temporally adjacent pixels are also covered by rain is negligible (Garg and Nayar, 2004). Therefore, we adopt *N*_*p*_ = 1 for rain streaks. Then, we have:


(2) $$r_{p}=\frac{1}{\bar{l}},$$

where $\bar{l}$ is the expected length of a rain streak and it is obvious that $\bar{l}\geq 1$. Therefore, (2) indicates that a raindrop or streak projects much fewer pixels in the W-T space.

Now, let us investigate how the number or density of rain streaks in one image changes in the W-T space. Given a rainy DVS event video $O\in {\mathbb{R}}^{h\times w\times t}$, then the average rain streak density in a frame of O can be computed as


(3) $$\begin{array}{r} \rho_{avg}=\frac{\bar{N}}{h\times w\times t}, \end{array}$$

where $\bar{N}$ is the quantity of total rain streak projections in O. In the W-T space, any transformed image Î_*i*_ is comprised of the *i*th row of several original images. We consider rain streaks are uniformly distributed in time and space. Then, the expected number of rain streaks that simultaneously cover the same row in the original image is:


(4) $$\begin{array}{r} n=\bar{N}/h\times t\times \bar{l}. \end{array}$$

Finally, the average rain streak density in a frame Î_*i*_ in the W-T space can be given as:


(5) $$\begin{array}{r} {\hat{\rho }}_{avg}=\frac{n\times t}{w\times t}=\frac{\bar{N}\times \bar{l}}{h\times w\times t} \end{array}$$

From (2), (3), and (5), one can make two conclusions:

- The number of pixels of raindrop or streak projects in the W-T space is $1/\bar{l}$ times that in the normal space.
- The density of rain streak projections in the W-T space is $\bar{l}$ times that in the normal space.

From the two observations, we find that in the W-T space one could “see” more “rain streaks” that are smaller in size than those from the original images. The rain streaks seem to be “ground” into approximately uniform noise by the transformation process. Due to the temporal continuity, the background object motions will be transformed into geometric curves that still be solid and have a regular appearance. Therefore, we can remove such rain streak noise and the system noise by denoising algorithms, which is the main motivation of our study. It is worth noting that the reason why we perform the deraining process in the width-time space instead of the height-time space is $\bar{l}>>\bar{b}$, i.e., we can get more outstanding “grinding” effect from the transformation process.

### 5.3. Example

**Figure 4:**
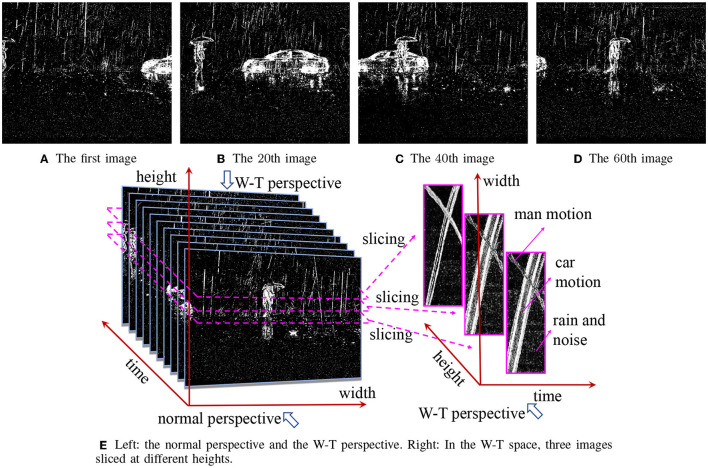
**(A–D)** Four frames from a rainy DVS sequence comprising 60 images. **(E)** Their W-T perspective.

In this example, we are given a DVS sequence comprising 60 images, in which a car moves from right to left and a man walks in an opposite direction. Figures 4A–D are four selected images. Please note that we select a sequence of 60 images only because it is long enough such that the images in the W-T space can be observed by naked eyes. Our approach needs just 5 frames to remove rains effectively.

The right part of Figure 4E shows three W-T space images, each image includes 2 evident curves with regular shapes, which is the motion of the car and the man in the original images. One can also find approximately uniform noise in the images. Recall the aforementioned intrinsic properties of rain streaks, we can recognize that the approximately uniform noise represents the raindrops and noise in the original images.

The background object motions are transformed into geometric curves that still have regular appearances while raindrops become approximately uniform noise which are much easier to remove by adopting state-of-art denoising methods. Therefore, we can remove raindrops and streaks in DVS videos in a simple but effective way, which is the main motivation of our study.

### 5.4. Remove Raindrops and Streaks

**Figure 5:**
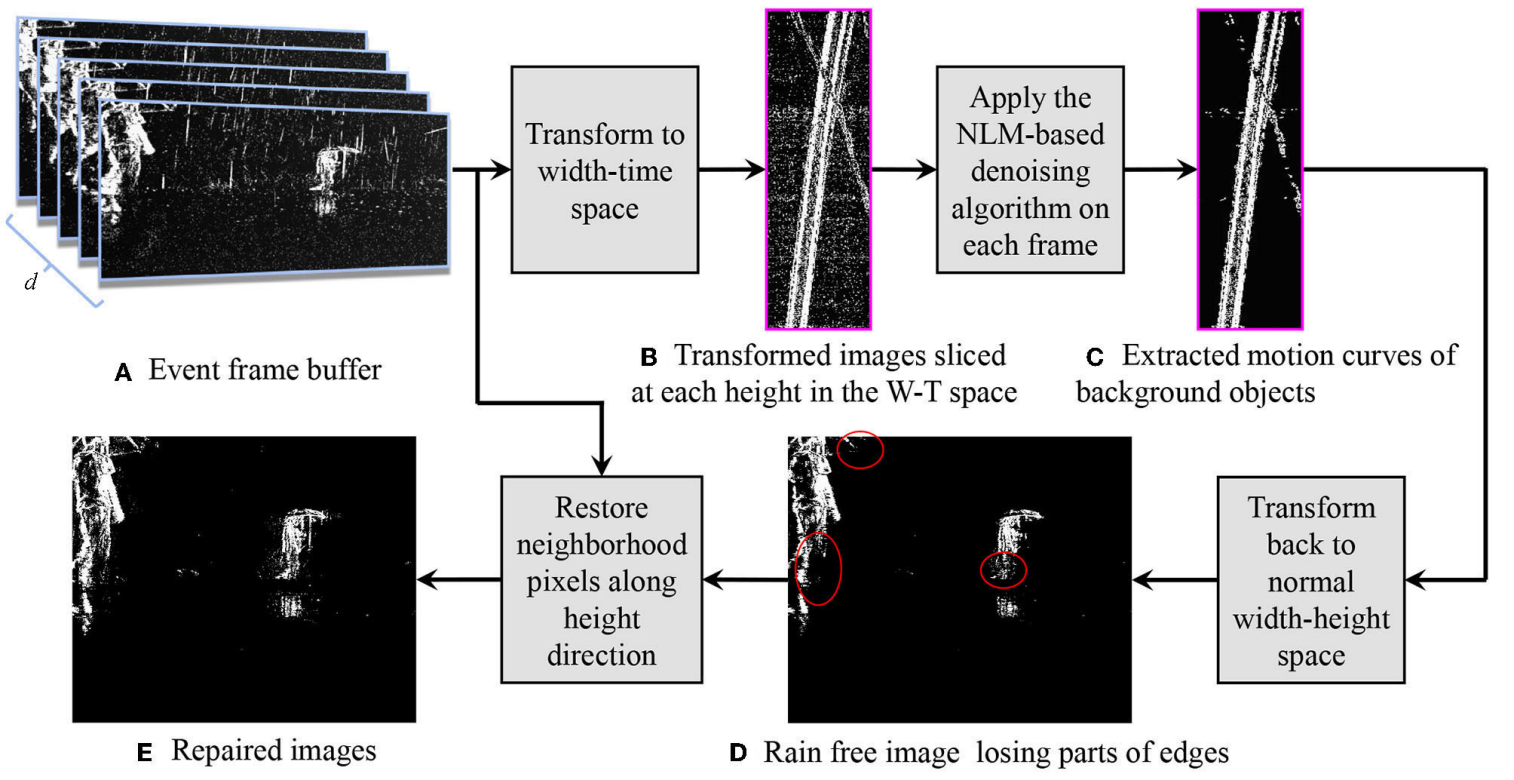
The structure of WTSD. **(A)** The event frame buffer. **(B)** A transformed image in the W-T space. Rain streaks and system noise become approximately uniform noise. **(C)** The extracted motion curves. Note that the edges are hampered. **(D)** After processing all the images in the W-T space, rain free images in normal space are retrieved by performing the transformation reversely. **(E)** Using our repairing method, we get images having more edge details.

Now, we discuss how to remove raindrops and streaks from DVS outputs based on the aforementioned principle. We name this method the Width and Time Space Deraining (WTSD) model, as shown in Figure 5.

A First-In First-Out buffer *B* (*h* × *w* × *d*) is created for the rain removal, and *d* is the buffer depth. Each layer of *B* comprises one normal event frame. The newest frame is pushed on the top of the buffer (layer 1) and the oldest frame is moved out from the bottom (layer *d*). The rest of the layers updates accordingly. Our rain removal approach works on the top layer frame (*I*_1_) for a quick response when implemented online.

The images in buffer *B* (*h* × *w* × *d*) are first transformed into the W-T space. Hence, the size of the image is *w* × *d* and the total number is *h*. In the W-T space, as aforementioned, the raindrops and original noise become approximately uniform noise while the object motions are transformed into thick solid curves. We observe that:

- The solid curves of object motions have periodic patterns. They have high texture similarities and similar pixels in these curves have no reasons to be close at all, as pointed out in Buades et al. (2011).
- The noise pixels transformed from raindrops and original noise have little correlation with each other.

Therefore, one could utilize pixel similarity as a criterion to distinguish object motions and raindrops. Due to the above reasons, the well-known non-local means (NLM) filter (Buades et al., 2011) is adopted to remove noise (raindrops and original noise) in the W-T space. With NLM, we can better remove the noise while preserving more details of background objects. Pseudo-codes of the noise removal algorithm are listed in Algorithm 1.

**Algorithm 1:** Remove rain and noise in the W-T space

**Input:** Source image ${\hat{I}}_{i}$, filter strength *H*, template window size *w*_*t*_, search window size *w*_*s*_, threshold *T*_*bin*_
**Output:** Denoised image ${Î}_{i}^{clean}$
get ${Î}_{i}^{nlm}$ by applying NLM filter on Î_*i*_ with parameters *H*, *w*_*t*_ and *w*_*s*_.
get ${Î}_{i}^{bin}$ by performing thresholding operation on ${Î}_{i}^{nlm}$ with threshold *T*_*bin*_.
${Î}_{i}^{clean}\leftarrow$ element-wise multiplication of Î_*i*_ and ${Î}_{i}^{bin}$.

Finally, combining the images in the W-T space and performing the transformation process reversely, we can reconstruct a rain free image $I_{1}^{clean}$ for the original image *I*_1_ in *B*. It must be noted that although the NLM successfully removes almost all the noise, it also aggressively erases several parts of the moving objects, especially the edges. Next, we discuss how to restore the lost information.

### 5.5. Repair

After performing the NLM-based denoising in the W-T space, we obtain an approximate binary mask of the background moving objects. Their shape and structure are correctly masked while details around edges and regions with discontinuous intensities may be erased. Considering such erased parts have a strong correlation with the extracted parts, we utilize the denoised image $I_{1}^{clean}$ and the original image *I*_1_ to restore the curves of motions.

In an image, a pixel *p* is located by a coordinate (*h*_*p*_, *w*_*p*_). The neighborhood of *p* is defined as a (2*r*+1) × 1 block:


(6) $$\begin{array}{r} H\left(p,r\right)=\left\{q\left(h_{q},w_{q}\right)|h_{q}\in \left[h_{p}-r,h_{p}+r\right],w_{q}=w_{p}\right\} \end{array}$$

Now, we simply replace the neighborhood pixels *H*(*p, r*) of a center *p*(*h*_*p*_, *w*_*p*_) by pixels in the same region in *I*_1_, if the pixel *p*(*h*_*p*_, *w*_*p*_) is activated in $I_{1}^{clean}$. Let $I_{1}^{rpd}$ denote the repaired image, we have:


(7) $$I_{1}^{rpd}(p)=\{\begin{array}{ll} I_{1}(p) & \text{if}\exists q\in H(p,r):I_{1}^{clean}(q)\geq 0\\ 0 & \text{otherwise} \end{array}$$

where *I*(*p*) means the value of pixel *p* in the image *I*.

This method can significantly restore the lost details at a light price of introducing tiny noise around background objects. Since the NLM filter almost removes all noise, adding such a tiny noise is acceptable.

## 6. Experiments

### 6.1. Setup

Since one can hardly find any generally accepted database for DVS deraining, we create a new real-world database that is recorded throughout our experiments. We name the database DVS-Deraining (DVS-D)^1^. The data is taken by a CeleX-IV DVS camera with the default configuration. The CeleX-IV is a sensor with 640 × 768 pixels and a maximum of 200 Meps output (Guo et al., 2017). We record the raw data from the sensor into a binary file, which comprises a series of stream events with each representing the full information of the fired pixels. The raw data can be decoded and visualized with different modes and parameters by the official software development kit (SDK) (CeleX4, 2019). A series of stream events of 26 s in length is selected as the input samples. All the experiments are conducted on a server of Ubuntu 18.04 with an Intel(R) Xeon(R) Gold 6134 CPU at 3.2 GHz, 8 × 32 GB RAM. Our approach is implemented in Python 3.6 and OpenCV 4.1. The NLM filter is configured as follows. The parameter regulating filter strength is set as *H* = 100, the template window size is 13, and the search window size is 15. The threshold in Algorithm 1 is 65.

We compare our approach (WTSD, the abbreviation for “Width and Time Space Deraining”) with three recent methods. They are the learning based deraining method named Recurrent Multi-scale Aggregation and Enhancement Network (ReMAEN) in Yang and Lu (2019)^2^; and Li M. et al. (2018)^3^; Multiscale Convolutional Sparse Coding (MCSC) method; and the method adopting the patch-based mixture of Gaussians (PMG) (Wei et al., 2017)^4^. In fact, ReMAEN is a single image based method while the other two methods are video based ones. However, as reported in Jiang et al. (2018), the learning based deraining methods could outperform some video based methods and show great vitality and a wide application prospect. Therefore, the comparison with ReMAEN is reasonable and challenging.

To evaluate these approaches by the same criteria, we select 50 frames from the inputs event video and then manually remove the rain streaks and noise to get a set of ground truth. The frames include scenes of “car and man”, “two walking men”, “single man: a distant view”, “single man: a close view”, and “only rain”. These 50 frames are used as the dataset or the testing set when comparing the approaches by quantitative indexes in Section 6.3. For method ReMAEN, another 200 different frames have also been labeled as the training set. For methods MCSC and PMG, the input images are first denoised by the mean filter to remove the system noise. Otherwise, both methods would be greatly disturbed by the system noise.

### 6.2. Visual Comparison

**Figure 6:**
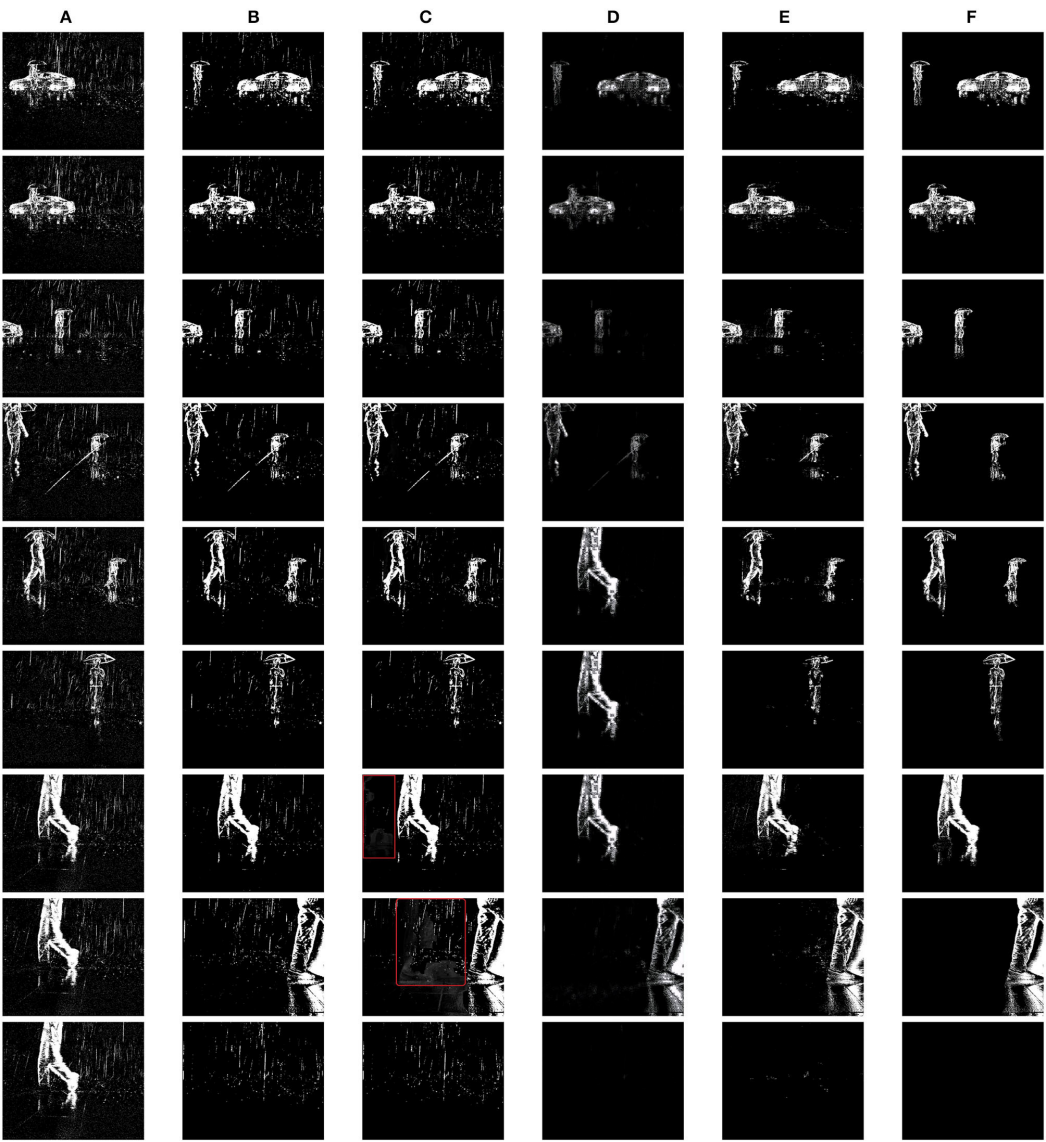
The rainy input, rain removal results by different methods, and the ground truth (GT) of nine different frames. WTSD is our approach while MCSC, PMG, and ReMAEN are the ones for comparison. Note the part framed in red of PMG results. **(A)** Input. **(B)** MCSC. **(C)** PMG. **(D)** ReMAEN. **(E)** WTSD. **(F)** GT.

Figure 6 shows the results of the four approaches conducted on 9 example frames. Our method utilizes the previous 10 frames to remove rain. One can observe that MCSC and PMG have a similar level of performance. They only remove part of the rain streaks from all input scenes due to that both approaches utilize the three-layer model to construct the image, i.e., the rain layer, background layer, and moving objects layer, while in DVS images only the rain and moving objects are presented. The low rank recovery-based optimization problems proposed by them may need reconstruction for DVS scenarios. Moreover, part local regions of certain images outputted from PMG get smeared due to the interference from temporal neighbor images. Examples are framed in red in Figure 6. Approach ReMAEN removes most of the rain streaks but hampers the texture details of background objects. In our approach, WTSD removes almost all the rain and noise in all scenes with a slight loss of background object details. In general, WTSD presents the best visual results compared to the ground truth.

### 6.3. Quantitative Results

For quantitative evaluation, we adopt a set of quantitative indexes, which are the mean squared error (MSE), the peak signal-to-noise ratio (PSNR), the visual information fidelity (VIF) (Sheikh and Bovik, 2006), the structural similarity (SSIM) (Wang et al., 2004), the multi-scale structural similarity (MS-SS) (Rouse and Hemami, 2008), the feature similarity (FSIM) (Zhang et al., 2011), and the universal image quality index (UIQI) (Wang and Bovik, 2002).

#### 6.3.1. Approaches Comparison

The performance indexes of all methods are calculated over the 50 frames. From the results presented in Table 1, one interesting observation is that the VIF indexes are low for all cases. The reason is that VIF assesses image quality mainly according to visual appearance. DVS images are actually the stream of events and their visual effect greatly differs from that of traditional images. Thus low VIF indexes are reasonable. ReMAEN and our approach have close PSNR, SSIM, and MS-SS indexes. The MS-SS from ReMAEN is even slightly better. It is in agreement with the aforementioned rationality of considering comparing with learning based single image deraining methods. Other indexes from ReMAEN are relatively worse because their training process is only evaluated by PSNR and SSIM indexes. In general, WTSD outperforms other compared methods in terms of most performance indexes, which confirms the conclusion from visual comparison.

**Table 1:** Quantitative comparison of different approaches.

**Method**	**MSE**	**PSNR**	**VIF**	**SSIM**	**MS-SS**	**FSIM**	**UIQI**
Input	2234.651	14.680	0.107	0.244	0.374	0.444	0.151
MCSC	1303.015	17.327	0.0574	0.823	0.755	0.756	0.787
PMG	1308.653	17.313	0.0546	0.801	0.746	0.738	0.680
ReMAEN	1045.639	20.267	0.10	0.906	**0.918**	0.765	0.401
WTSD	**790.034**	**20.782**	**0.262**	**0.922**	0.904	**0.922**	**0.917**

*Values in boldface are the best ones*.

#### 6.3.2. Parameters

We report how our approach performs with different parameters. Note that we only change one parameter at a time and the others are set as their defaults, which are: *d* = 10, *h* = 640, *w* = 768 and the event frame time *T*_*f*_ = 30 ms.

**Event Buffer Depth** We vary the buffer *B* depth *d* from 3 to 50 and run our approach for the 50 frames.

From Table 2, one can observe that although our approach generally performs best for a depth of 50, it can still provide close SSIM and UQI results and even better MSE, PSNR and MS-SS results for a depth of 5. Therefore, our approach can work properly with a small volume of historical data. This makes the online implementation possible in terms of both computation and storage requirements. The best results of MSE and PSNR are obtained for a depth of 3. The reason is the NLM window size is far larger than the depth. This leads to minimal edge detail loss, which can compensate for the MSE and PSNR degradation caused by a few left rain streaks. It is worth noting that we still get acceptable results for the other depths, which indicates the robustness of the approach.

**Table 2:** Quantitative comparison of different event frame buffer depths.

**d**	**MSE**	**PSNR**	**VIF**	**SSIM**	**MS-SS**	**FSIM**	**UIQI**
3	**469.267**	**21.801**	0.209	0.891	0.878	0.852	0.902
5	744.283	20.846	0.229	0.920	**0.908**	0.913	0.916
7	818.348	20.612	0.233	0.917	0.903	0.919	0.913
10	846.601	20.577	0.243	0.920	0.904	0.923	0.915
15	840.685	20.606	0.244	0.920	0.905	0.923	0.916
20	840.685	20.606	0.244	0.920	0.905	0.923	0.916
25	840.685	20.606	0.244	0.920	0.905	0.923	0.916
30	835.386	20.670	0.246	0.921	0.905	0.923	0.916
40	822.589	20.765	0.249	0.921	0.906	0.924	0.916
50	810.712	20.830	**0.253**	**0.922**	0.907	**0.925**	**0.917**

*Values in boldface are the best ones*.

**Event Frame Time** In the *Event Data* mode, the events are grouped as event frames and outputted periodically. For a smaller event frame time, the rain streaks are shorter. Now, we study how our approach handles such variations of raindrops and streaks. The inputs are re-generated from the raw data using the official SDK for event frame times [5, 15] ms. Since the number of frames rapidly increases when *T*_*f*_ gets smaller, manually removing rain for *T*_*f*_ = 5 ms becomes a task that is almost impossible for us to complete. Hence, we only compare the results of five time instances with a buffer depth of 10. The results are presented in Table 3.

**Table 3:** Quantitative comparison of different event frame times *T*_*f*_ (ms).

***T*_*f*_**	**MSE**	**PSNR**	**VIF**	**SSIM**	**MS-SS**	**FSIM**	**UIQI**
30	646.495	20.461	**0.304**	0.933	0.913	0.937	0.929
15	477.290	21.707	0.302	**0.951**	**0.938**	0.962	**0.941**
5	**298.897**	**23.948**	0.181	0.942	**0.938**	**0.964**	0.927

*Values in boldface are the best ones*.

The overall best performance is produced when *T*_*f*_ = 15 ms. The reason is that the discontinuity of rain in the time direction is more evident due to shorter streaks. Note that only the VIF index gets a notable degradation when *T*_*f*_ = 5 ms. This can be explained as the continuation of background objects is also hampered for small *T*_*f*_, which may cause damage to the object structure. The values of MSE and PSNR are the best when *T*_*f*_ = 5 ms, this can be interpreted for a similar reason as the previous section.

#### 6.3.3. Discussion of Each Component

We study the distinctive effects of the Our approach has two major components: the NLM-based denoising and the repairing processes.

To elaborate on their distinctive effects of them, we substitute two classical denoising methods, i.e., the median filter (MF) and the side window median filter (SWM) (Yin et al., 2019)^5^, for the non-local means filter and then test them with and without the repairing process. We also report their results when all the frames are put in buffer *B*, i.e., the case that performing offline global deraining for the whole video. All the denoising methods are applied in the W-T space. The results are depicted in Table 4.

**Table 4:** Quantitative comparison when adopting different denoising methods in the W-T space.

**Method**	**MSE**	**PSNR**	**VIF**	**SSIM**	**MS-SS**	**FSIM**	**UIQI**
NLM	846.601	20.577	0.243	0.920	0.904	0.923	0.915
NLM-R	790.034	20.782	0.262	0.922	0.904	0.922	0.917
NLM-RG	**379.717**	**23.185**	**0.369**	**0.947**	**0.933**	**0.942**	**0.936**
MF	795.768	19.433	0.0837	0.828	0.810	0.751	0.857
MF-R	762.147	19.518	0.102	0.831	0.789	0.745	0.857
MF-RG	597.407	20.808	0.151	0.900	0.860	0.860	0.889
SWM	1325.803	17.001	0.0779	0.648	0.598	0.615	0.146
SWM-R	2221.985	14.705	0.106	0.246	0.375	0.445	0.154
SWM-RG	995.026	18.348	0.133	0.649	0.660	0.632	0.553

*NLM-R denotes the NLM-based method for repairing. NLM-RG means the same method when adopting global deraining. Same notations are applied to the MF and SWF-based methods. Values in boldface are the best ones*.

As the results show, the NLM-based methods (NLM-RG) outperform others. Observe the performance is improved when the repairing process joins. It is worth noting that using global deraining leads to the best result for every method. This is expected since we can utilize the maximal amount of history and future data to remove noise in the W-T space for most images. Therefore, the global deraining approach (NLM-RG) is suitable for offline implementation for optimal performance.

### 6.4. Potential Implementation for Classical Images

**Figure 7:**
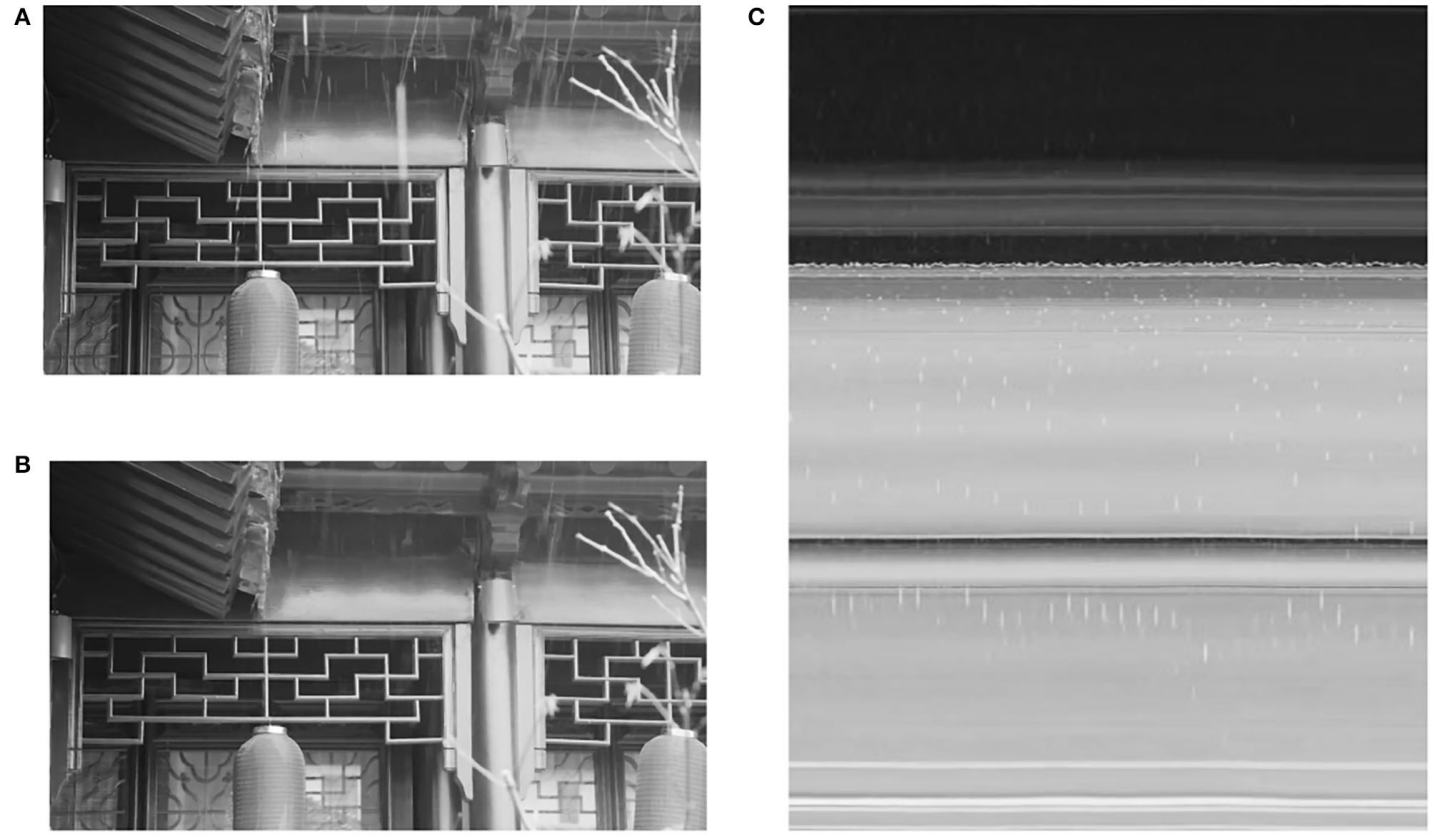
**(A)** One image selected from a normal rainy video. **(B)** The corresponding output image from our approach. **(C)** One image in the W-T space sliced at a height of 200.

We have demonstrated that by transforming DVS images into the W-T space, almost all rain streaks from a DVS camera can be removed. Finally, we present an example of the implementation of the W-T space for traditional cameras. In the denoising process, the NLM filter is replaced by the SWM filter to preserve edge information in the images. Moreover, the repairing component is skipped since it is designed for DVS images. As shown in Figure 7, in the W-T space, one can observe the noise transformed from rain streaks and also the curves having regular patterns. Note that in the output image, most rain streaks are removed while the image is slightly blurred. We can further exploit the regular patterns of the background to keep more details in the W-T space, which shall be our future study.

## 7. Conclusion

In this article, we have presented a novel, simple but surprisingly effective approach to removing raindrops and streaks from a DVS event video. Based on the prior that rain is discontinuous in the width and time directions, we propose to perform the deraining process in the width and time space, in which rain appears to be approximately uniform noise that can be easily removed. We find that the non-local means filters produce good deraining results when adopt in the denoising process in the W-T space. The experimental results demonstrate that our approach can better remove rain noise than the four existing methods for traditional camera videos. The best performance is achieved when the event buffer depth is 50 and the event frame time is 15 ms. We also create a new real-world database for deraining algorithms for DVS images. In summary, we have made the following findings in this manuscript.

- The output of DVS has two unique features: (1) no stationary information and (2) no grayscale and RGB information. These make conventional deraining methods no longer suitable for DVS.
- The number of pixels of raindrop or streak projects in the W-T space is $1/\bar{l}$ times that in the normal space.
- The density of rain streak projections in the W-T space is $\bar{l}$ times that in the normal space.
- In the denoising process in the W-T space, the non-local means filter produces good deraining results as background object motions have periodic patterns.
- The parameter event buffer depth has little influence on the performance.
- Our approach also has the potential to handle traditional RGB rainy videos, which is our future study.

## Data Availability Statement

The raw data supporting the conclusions of this article will be made available by the authors, without undue reservation.

## Author Contributions

LC: methodology, investigation, software, writing—original draft, and writing—review and editing. NL: software and validation. XG: formal analysis and review. YS: conceptualization and formal analysis. ZM: resources and review. KH: conceptualization and resources. XZ: review, project administration, and funding acquisition. All authors contributed to the article and approved the submitted version.

## Funding

This study has been partly funded by Nation Natural Science Foundation of China (Grant No. 61902442), the National Social Science Fund of China (Grant No. 21ZDA028), and Guangdong International Cooperation Program with No. 2021A0505030024.

## Conflict of Interest

The authors declare that the research was conducted in the absence of any commercial or financial relationships that could be construed as a potential conflict of interest.

## Publisher's Note

All claims expressed in this article are solely those of the authors and do not necessarily represent those of their affiliated organizations, or those of the publisher, the editors and the reviewers. Any product that may be evaluated in this article, or claim that may be made by its manufacturer, is not guaranteed or endorsed by the publisher.

## Footnotes

^1^ https://github.com/chenglong3383/DVS-D

^2^ https://github.com/nnUyi/ReMAEN

^3^ https://github.com/MinghanLi/MS-CSC-Rain-Streak-Removal

^4^ https://github.com/wwzjer/RainRemoval_ICCV2017

^5^ https://github.com/Ldpe2G/ArmNeonOptimization/tree/master/sideWindowBoxFilter
